# Stroke follow-up in primary care: a Norwegian modelling study on the implications of multimorbidity for guideline adherence

**DOI:** 10.1186/s12875-019-1021-9

**Published:** 2019-10-18

**Authors:** Rune Aakvik Pedersen, Halfdan Petursson, Irene Hetlevik

**Affiliations:** 1 General Practice Research Unit, Department of Public Health and Nursing, NTNU, Norwegian University of Science and Technology, PO Box 8905 MTFS, N-7491 Trondheim, Norway; 2 Research and Development Primary Health Care, Research and Development Center Gothenburg and Södra Bohuslän, Region Västra Götaland, Sweden

**Keywords:** Stroke, General practice, Practice guidelines, Multimorbidity

## Abstract

**Background:**

Specialized acute treatment and high-quality follow-up is meant to reduce mortality and disability from stroke. While the acute treatment for stroke takes place in hospitals, the follow-up of stroke survivors largely takes place in general practice. National guidelines give recommendations for the follow-up. However, previous studies suggest that guidelines are not sufficiently adhered to. It has been suggested that this might be due to the complexity of general practice. A part of this complexity is constituted by patients’ multimorbidity; the presence of two or more chronic conditions in the same person. In this study we investigated the extent of multimorbidity among stroke survivors residing in the communities. The aim was to assess the implications of multimorbidity for the follow-up of stroke in general practice.

**Methods:**

The study was a cross sectional analysis of the prevalence of multimorbidity among stroke survivors in Mid-Norway. We included 51 patients, listed with general practitioners in 18 different clinics. The material consists of the general practitioners’ medical records for these patients. The medical records for each patient were reviewed in a search for diagnoses corresponding to a predefined list of morbidities, resulting in a list of chronic conditions for each participant. These 51 lists were the basis for the subsequent analysis. In this analysis we modelled different hypothetical patients and assessed the implications of adhering to all clinical guidelines affecting their diseases.

**Result:**

All 51 patients met the criteria for multimorbidity. On average the patients had 4.7 (SD: 1.9) chronic conditions corresponding to the predefined list of morbidities. By modelling implications of guideline adherence for a patient with an average number of co-morbidities, we found that 10–11 annual consultations with the general practitioner were needed for the follow-up of the stable state of the chronic conditions. More consultations were needed for patients with more complex multimorbidity.

**Conclusions:**

Multimorbidity had a clear impact on the basis for the follow-up of patients with stroke in general practice. Adhering to the guidelines for each condition is challenging, even for patients with few co-morbidities. For patients with complex multimorbidity, adhering to the guidelines is obviously unmanageable.

## Background

### Stroke follow-up in general practice

Each year about 15,000 persons suffer a stroke in Norway. The acute treatment most often takes place in specialized stroke-units in hospitals, but the follow-up of survivors residing in the communities takes place in general practice. This is in accordance with the national guidelines for treatment of stroke [1] which state that general practitioners (GPs) should play a key role in the follow-up of stroke survivors. All residents in Norway are entitled to a regular general practitioner (RGP) and at the time of the study, about 99% of the Norwegian population were registered on RGP’s lists [2].

The guidelines give normative advice on the contents of the follow-up in general practice. They recommend that patients with stroke should normally be given lipid-lowering treatment in the form of statins. Creatine kinase (CK) and transaminase blood samples should be taken to control possible side-effects of this medication. The target value for low-density lipoprotein (LDL) should be < 2.0 mmol/L, and the target value for blood pressure should be < 140/90 mmHg. Diet, body mass index (BMI), physical activity, alcohol consumption, and smoking affect the risk for stroke and recurrent stroke. These lifestyle factors should therefore also be part of the post-stroke follow-up. However, an increasing amount of evidence suggests that the follow-up in general practice is not in accordance with clinical guidelines [3–7]. This includes previous analysis of data on the same participants as the present study, where we found that most consultations with the RGP the first year after a stroke were concerned with issues other than the stroke, and that guidelines were often not adhered to [3]. Multiple factors can explain non-adherence to clinical guidelines, such as lack of familiarity with the recommendations, but complexity of patient situations has also been identified as a barrier to the implementation of clinical guidelines [8, 9].

### Multimorbidity

There is no international consensus on a standardized list of chronic conditions or a standard for the measurement of multimorbidity [10–13]. Therefore there is a need to operationally define which conditions to include in a multimorbidity count [14]. Definitions of multimorbidity vary in the number and kinds of conditions included. Most often, multimorbidity is defined as the presence of two or more chronic medical conditions in the same person [12, 13, 15–17]. Recent publications point out that the GPs are situated in a landscape that is more complex than what is reflected by organ-specific guidelines, and that this landscape is dominated by multimorbidity. According to Tomasdottir et al. “the disease clusters typically transcend biomedicine’s traditional demarcations between mental and somatic diseases and between diagnostic categories within each of these domains” [18]. In general practice, multimorbidity is the rule rather than the exception [15, 18, 19].

Multimorbidity poses a challenge to patient safety, in part due to the complex management regimens [20]. It has been documented that when the treatment of patients who have multiple concurrent diseases is in accordance with the relevant guidelines, this can give unfortunate results [21, 22]. An example is polypharmacy with significantly increased risk of drug side effects and interactions [23]. Hence, GPs can experience situations where adherence to guidelines is incompatible with a patient-centered approach to the patient with multimorbidity [24]. Furthermore, in the presence of multiple coexisting conditions, the benefits and harms associated with the combination of recommended treatments become unclear and priorities become uncertain [25]. Multimorbidity and polypharmacy have been documented to be more common among persons with stroke than those without [26].

The aim of this study was to assess the implications of multimorbidity on the follow-up of stroke in general practice. More detailed aims were:

- To investigate the extent of multimorbidity among patients who had suffered an ischemic stroke.
- To map the most common co-morbidities.
- To estimate the annual number of guideline-recommended investigations and follow-up visits to the GP or other healthcare providers for a stroke survivor with a typical combination of chronic conditions.

## Methods

This study was designed as a cross-sectional analysis of prevalence of multimorbidity in patients with stroke in Norway, and assessment of the implications of adherence to clinical guidelines. We used the STROBE statement [27] to guide our reporting of the study. Multimorbidity was defined as the presence of two or more chronic medical conditions in the same person. A pre-specified list of 40 conditions (see: Additional file 1), developed by Barnett and colleagues [11], was used as a frame for the morbidity-count. We included patients treated for ischemic stroke in two local hospitals in Mid-Norway in 2011 and 2012. All patients with the discharge diagnosis I63.0 trough I63.9 according to the International Statistical Classification of Diseases and Related Health Problems, 10th revision (ICD-10), were identified in the hospital files. The Norwegian Health Economics Administration identified the RGP for each of the patients. Each of these GPs were invited to participate. Subsequently, identified stroke patients were invited if they were living in their own home and registered with an RGP who had accepted participation. Patients in nursing homes were excluded.

### Data collection

One of the authors (RAaP) personally visited each clinic and reviewed the continuous text of the medical records, the diagnosis records, laboratory records, and the prescribing registries for each individual patient. Diagnoses that met the pre-specified diagnostic criteria (Additional file 1) were registered, resulting in a list of chronic conditions for each participant.

### Analysis

The number of chronic conditions was counted for each participant and the frequency of each condition registered (Table 1).

**Table 1:** Co-existing chronic conditions among the 51 patients with stroke

Condition	N	%
Stroke	51	100
Hypertension	28	55
Coronary heart disease	24	47
Rheumatoid arthritis, other inflammatory polyarthropathies & systematic connective tissue disorders	13	25
Diabetes	11	22
Atrial fibrillation	10	20
Prostate disorders	9	18
Hearing loss	9	18
Treated dyspepsia	8	16
Anxiety & other neurotic, stress related & somatoform disorders	7	14
Asthma	7	14
Painful condition	7	14
Depression	6	12
Chronic obstructive pulmonary disease	6	12
Blindness & low vision	6	12
New diagnosis of cancer in the last 5 years	5	10
Epilepsy	5	10
Thyroid disorders	4	8
Chronic kidney disease	4	8
Peripheral vascular disease	3	6
Heart failure	3	6
Alcohol problems	2	4
Migraine	2	4
Psoriasis	2	4
Diverticular disease of intestine	2	4
Learning disability	1	2
Inflammatory bowel disease	1	2
Chronic sinusitis	1	2
Other psychoactive substance misuse	1	2

To assess the implications for the follow-up of stroke, we constructed three follow-up situations, representative for the study population, that typical patients would find themselves in if all isolated conditions were to be followed up according to “best practice”, i.e., in accordance with all relevant guidelines. The constructions were hypothetical examples representative regarding number and type of chronic condition. Hypothetical rather than real patients were chosen to eliminate the risk of identification of specific participants. First, we defined age, gender and number of chronic conditions for the hypothetical patients. To reflect the different grades of multimorbidity among the patients, we chose different numbers of conditions for each of the examples. The number of conditions for each example was selected based on the spectrum we found among the participants. The first example represented the patients with the least complex multimorbidity among the stroke survivors (Example 1: a patient with three morbidities including stroke). The number of chronic conditions for this example was below average. The second example represented an average number of chronic conditions (Example 2: a patient with five morbidities including stroke). The third example represented the patients with the most complex multimorbidity, with a number of chronic conditions above average (Example 3: a patient with seven morbidities including stroke). For each example we chose the defined number of conditions among the 20 most frequent conditions (Table 1). In this way, only conditions affecting several patients in our study were taken into account.

Only conditions with national clinical guidelines or similar formal recommendations were selected. Recommendations on follow-up were extracted from relevant guidelines and the number of recommended follow-ups with the GP and organ-specific specialists was registered into a table for each example (Tables 2, 3 and 4). Recommendations regarding treatment by other health care providers, laboratory tests and special procedures were also recorded.

**Table 2:** Patient 1: Recommended annual follow-up activity

	Consultations with the GP	Consultations with specialists	Other recommended health care providers	Laboratory tests	Special Procedures
COPD	1–2	NR	Physiotherapist (limited to 40 annual treatments), two supervised work-outs a week	NR	Spirometry Vaccination
Colorectal cancer	2	NR	NR	yes	2 x CEUS and 1 x LDCT
Smoking	4	NR	NR	NR	NR
Driver’s licence	1	NR	NR	NR	NR
Stroke	1	1	NR	yes	NR
Total	9–10	1	yes	yes	5

Minimum follow-up activity recommended for a period of 12 months, given that all conditions are clinically stable, and no new abnormalities are found in the tests

Abbreviations: *COPD* Chronic obstructive pulmonary disease, *CEUS* Contrast-enhanced ultrasonography, *LDCT* Low-dose computed tomography, *NR* No recommendations

**Table 3:** Patient 2: Recommended annual follow-up activity

	Consultations with the GP	Consultations with specialists	Other recommended health care providers	Laboratory tests	Special procedures
Asthma	1	NR	physiotherapist		Spirometry
Diabetes	2	1–2	NR	yes	NR
RA	4	1	NR	yes	NR
Thyroid disorder	2	NR	NR	yes	NR
Screening	0–1	NR	NR	yes	Gynecological examination. Mammography
Stroke	1	1	NR	yes	NR
Total	10–11	3–4	yes	yes	3

Minimum activity recommended for a period of 12 months for the patient in example 2, given that all conditions are clinically stable, and no new abnormalities are found in the tests

Abbreviations: *RA* Rheumatoid arthritis, *NR* No recommendations

**Table 4:** Patient 3: Recommended annual follow-up activity

	Consultations with the GP	Consultations with specialists	Other recommended health care providers	Laboratory tests	Special procedures
Diabetes	2	1–2	NR	yes	
COPD	1–2	NR	Physiotherapist (limited to 40 annual treatments), two supervised work-outs a week	NR	Spirometry. Vaccination
Colorectal cancer	2	NR	NR	yes	2 x CEUS and 1 x LDCT
Depression	6	NR	NR	NR	NR
Painful condition	6	NR	NR		NR
Thyroid disorder	2	NR	NR	yes	NR
Screening	0–1	NR	NR	yes	Gynecological examination. Mammography
Smoking	4	NR	NR	NR	NR
Stroke	1	1	NR	yes	NR
Total	24–26	2–3	yes	yes	7

Minimum activity recommended for a period of 12 months for the patient in example 3, given that all conditions are clinically stable, and no new abnormalities are found in the tests

Abbreviations: *COPD* Chronic obstructive pulmonary disease, *CEUS* Contrast-enhanced ultrasonography, *LDCT* Low-dose computed tomography, *NR* No recommendations

## Results

We identified 414 patients with the discharge diagnosis I63.0 trough I63.9 according to ICD-10 in the hospital files. They were listed with 100 different GPs. Among 100 invited GPs, 37 in 18 different clinics agreed to participate. In total 138 patients were invited to participate in the study, 51 gave their written consent and were included. Thirty (59%) were male and 21 (41%) were female, aged 38 to 90 years (mean 68.5 years).

With the range of 2–10 chronic conditions, all participants met the criteria for multimorbidity. On average the patients had 4.7 (SD: 1.9) chronic conditions corresponding to the list of 40 (Additional file 1), stroke included.

### Analysis of the health care burden

**Fig. 1:**
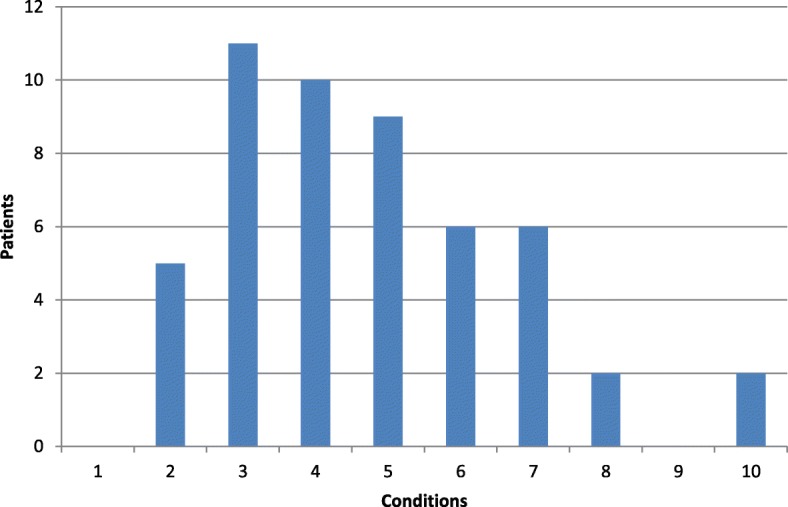
shows the distribution of patients with 0–10 chronic conditions. The number of patients with 0–10 chronic conditions.

Among the participants, 46 (90.2%) had three or more morbidities (see Fig. 1). Ten (19.6%) had seven or more morbidities. In the first example, we chose three chronic conditions including the stroke. This is about one standard deviation (SD) below average.

#### Example 1: a patient with three morbidities including stroke

A male smoker, 74 years, recently suffered a minor stroke with full recovery. He has chronic obstructive pulmonary disease (COPD) and 1 year before the stroke, he was treated for colorectal cancer. The cancer treatment was curative. He is now motivated to cease smoking.

Norwegian national guidelines for the follow-up of patients with COPD [28] were issued in 2012. It is recommended that patients with stable mild or moderate COPD have follow-up consultations with their GPs at least once a year, minimum twice if the COPD is severe. Annual controls should include spirometry, body mass index (BMI) registration, measurement of oxygen saturation, grading of dyspnea according to the British Medical Research Council (BMRC) scale, COPD questionnaire, mapping of physical activity, mapping of the number of exacerbations, evaluation of comorbidities, evaluation of each of the prescribed drugs, assessment of the need for rehabilitation, assessment of the need for specialized healthcare and advice on vaccination.

In the case of hospitalization it is recommended to have an additional consultation within 4 weeks of discharge. Smokers should be encouraged to cease smoking, motivation should be explored and help to cease smoking offered at every suitable consultation. Orientation on medication aided smoke cessation should be given. If motivated for cessation, the patient should be followed-up closely the first months. If cessation is initiated at the hospital, the GP must be involved by making an appointment for follow-up.

Patients with moderate to severe COPD should be referred to a physiotherapist for exercises regarding muscular strength, endurance, mobility, training in breathing techniques and secretive mobilization techniques. The patient should work out at least three times a week, two of which under supervision. The national health insurance scheme supports up to 40 annual treatments with physiotherapist. An annual influenza vaccination should be given.

There are national guidelines for the follow-up of colorectal cancer [29]. These guidelines provide an established form for the follow-up. The controls are performed by the patient’s GP, but the first check after surgery is to be performed by a surgeon. The second year of follow-up includes carcinoembryonic antigen (CEA) blood samples at 18 and 24 months after the surgery. Every 6 months a contrast-enhanced ultrasonography (CEUS) of the liver is to be performed. A low-dose computed tomography (LDCT) of the thorax is recommended every 12 months.

The national guidelines for smoking cessation [30] recommend that a structured aid for smoking cessation should include at least four meetings or consultations with the addition of follow-up according to need.

To continue driving at the age of 75, a person in Norway must undergo a health check with the GP. A person who has suffered a stroke can meet the health requirements after 3 months provided complete recovery. This can be assessed by the specialist at an outpatient control after the stroke, but an assessment of the combined health requirements for the driver’s license is more comprehensive and includes all aspects of health with potential impact on road safety. There is a separate form for the health certificate [31] and the health requirements for driver’s license are specified in guidelines issued in 2016 [32].

#### Example 2: a patient with five morbidities including stroke

A 68-year-old woman recently suffered a stroke. She also has the combination of thyroid disorder, asthma, type 2 diabetes and rheumatoid arthritis (RA).

The second example represents the average stroke survivor residing in the community.

Norwegian guidelines for asthma in general practice were issued in 2015 [33]. According to these, asthma is to be checked in an annual control. This control should include lung function measurements with a liberal use of reversibility testing, referral to physiotherapist and an assessment of the need for referral to a specialist or to a rehabilitation institution. Newly diagnosed patients should normally come to a control within 3 months after the first consultation and thereafter every 3–6 months. After an exacerbation, it is important to offer follow-up after 2–4 weeks. For patients with stable and good disease control, follow-up once a year is considered sufficient.

The national guidelines for diabetes [34] recommend one extensive control annually with the GP. Between the annual controls it is recommended to have at least one consultation for diabetes if it is well-regulated. More if needed. Patients with type 1 diabetes should in addition have interdisciplinary follow-up in the specialist health service at least once a year, patients with type 2 diabetes should be referred to an interdisciplinary team in the specialist health service in the case of coexisting complicating disease. The patient should be referred to an ophthalmologist at the time of diagnosis. If there is no sign of retinopathy, controls every 2 years is sufficient.

Regarding the RA, there is currently no national guideline for the follow-up in general practice, but the University hospital for the study region, St. Olavs Hospital in Trondheim, has issued recommendations for the follow-up in general practice [35], and these recommendations are published online at legehandboken.no, an evidence-based clinical decision support online resource used by more than 90% of Norwegian GPs [36]. Patients with disease modifying antirheumatic drugs (DMARDs) or biological medication are to be checked at the hospital’s outpatient clinic annually, and if necessary, more often. The GP should conduct a regular clinical examination with joint examination and blood pressure measurement. It is important to be aware of infectious diseases, and in the case of persistent airway symptoms, chest x-ray and spirometry should be taken. Laboratory tests should be taken every third month when clinical presentation and laboratory tests are stable.

Advice on follow-up of thyroid disorders is also available online in evidence-based clinical decision support resources. Patients with maintenance treatment need follow-up 1–2 times a year, more often when medication is adjusted.

Women in Norway are at the time of this study generally recommended to follow the cervical cancer screening program with screening every third year through age 69 years and the breast cancer screening program with screening every second year through age 69 years. The breast cancer screening takes place in radiology departments and does not necessarily involve the GP. The cervical cancer screening involves a gynecological examination, usually performed by the GP.

#### Example 3: a patient with seven morbidities including stroke

A 65-year-old woman who recently suffered a stroke. 2 years ago, she was curative treated for colorectal cancer. She has type 2 diabetes, COPD, a painful condition in the back, thyroid disorder and she is mildly depressed.

In the third example, we chose seven chronic conditions, including stroke. This corresponds with one SD above average.

The national guidelines for use of opioids with long lasting non-malignant pain [37] recommends careful follow-up in general practice. Patients with opioids for non-malignant pain should have control appointments with the GP at least every second month. The aim of these consultations is to control and prevent side-effects such as addiction and obstipation.

In Norway, a clear majority of patients with depression have their treatment exclusively in primary care. This is mainly people suffering from mild to moderate depression [38]. National guidelines for treatment of adults with depression states that these patients may benefit from short-treatment in primary care. It is recommended to consider counseling in relation to everyday problems, short-term cognitive therapy or interpersonal counseling with six to eight treatments over a period of 10 to 12 weeks. Antidepressants should be considered if the depression does not respond to non-medication attempts [38].

## Discussion

With a mean of 4.7 (SD: 1.9) chronic medical condition, none of the participants of the study had fewer than two morbidities, including stroke. Hypertension, coronary heart disease, rheumatic diseases as a group, and diabetes being the most prevalent co-morbidities. Multimorbidity had a clear impact on the basis for the follow-up of patients with stroke in general practice. We found that the overall follow-up regimen implicated by the different guideline recommendations can be challenging even for those patients in our study who had the fewest co-morbidities. Norwegian GPs find the workload heavy and increasing. Concern is expressed that this may compromise patient safety and recruitment of GPs [39]. In this context, the total regimen for the patients with the most complex multimorbidity is evidently unmanageable for the GPs. It must also be overwhelming for the patients.

We found that a high annual number of consultations with the GP were required for patients with multimorbidity, according to guideline recommendations. This cannot necessarily be solved by doing several things at the same time or in the same consultation, as the consultations are time-limited. In Norwegian general practice, a consultation is normally limited to 15–20 min [40]. Some of the procedures recommended by guidelines are so time consuming that there is hardly sufficient time for one procedure in the consultation. The annual diabetes control is an example of such a time consuming procedure [34]. Multimorbidity adds to the complexity of the consultations the increased risk of drug-drug and drug-disease interactions [41].

Our findings represent the recommendations provided that all conditions are clinically stable, and no new abnormalities are found in the tests. It is foreseeable that all conditions in patients with multimorbidity are not clinically stable along the timeline, this is a logical foundation for the guidelines. Abnormal findings in tests, clinical exacerbations of chronic conditions, or intercurrent diseases along the timeline will necessitate further procedures and hence further consultations.

### Findings in the light of current knowledge

In a previous study [3], we have shown that stroke survivors residing in the communities often consult their GPs, but also that adherence to guidelines for stroke follow-up [1] is limited. Multimorbidity is a part of the complexity of general practice, and findings in our present study provide an explanation of why the combined recommendations of guidelines may be too challenging to adhere to.

The complex topic of the doctor-patient relationship is well described in several dimensions. Structure, communication, and patients’ perspectives have been among the areas of research [42–44]. Time constraints has been identified among the systemic factors that affect this relationship [45].

There is no tradition for the use of modelling studies in assessing the consequences of guideline development before implementation is initiated. However, research of this kind has documented that guideline implementation can destabilize the health care service. For example, the monitoring and follow-up of blood pressure according to international expert guidelines may alone require more resources than available in general practice [46].

While much is known about what constitutes a good doctor-patient relationship, little is known about the capacity of this relationship. There must exist some limit to the extent of follow-up in general practice. We suggest that this limit is determined by the capacity of the doctor-patient relationship. This is obviously no fixed entity. It must depend on the patient capacity combined with that of the patient’s GP. With the term patient capacity, we mean the patient’s willingness and ability to participate in consultations, procedures, examinations and treatments. The doctor capacity may be determined by workload among other possible factors. We have no measure for the capacity of the doctor-patient relationship in general practice. However, a previous study by our research group showed that stroke survivors on average consulted their GPs 7.5 times the first year after the stroke [3]. This is not sufficient to control the stable state of the morbidities of any of our example-patients if the guidelines were adhered to. The patient in example 1 had below average complexity and the patient in example 2 had the same level of complexity as the average stroke survivor. This fact may indicate that the capacity of the doctor-patient relationship is exceeded even among those patients with the least co-morbidities. Simplifying treatment regimens as a strategy for safer care for people with multimorbidity has been previously suggested [20], and the findings in our study adds to the knowledge supporting such a view. More resources might solve problems related to doctors’ capacity, but they would not necessarily solve problems related to patients’ capacity.

Guidelines are usually developed according to international standards [47]. Our findings point out a substantial weakness in the guideline development. Their combined recommendations for the follow-up are not sustainable when it comes to patients with multimorbidity. As multimorbidity is the rule rather than the exception in general practice [15, 18, 19], guidelines, at least in Norway, are poorly adapted to patients’ clinical reality even if they comply with Norwegian guidelines for guidelines [48]. It has previously been raised critical questions as to whether the theoretical basis for the guidelines is good enough [5]. The findings in this study show that such questions are still relevant.

The general practice perspective tends to be inadequately addressed in guidelines, with factors such as workload and resources insufficiently taken into account. Partly, we believe this is due to inadequate involvement of GPs in guideline panels. Furthermore, recommendations on the frequency of follow-up visits is usually based on expert opinion, as there is rarely any direct evidence available to support these recommendations [1, 38].

We recommend future research to further explore undesired consequences of adherence to clinical guidelines in general practice. We also recommend the theoretical basis for guideline development to be scrutinized.

### Strengths and limitations

With the aim to assess the possible implications of multimorbidity for the follow-up of stroke in general practice, we see it as a major strength that we analyzed data from the GPs’ own documentation, rather than self-reported disease counts, for instance. In this way, we could assess the extent of multimorbidity from the GP’s point of view. All data collection was done by the same person, eliminating the risk of inter-observer differences in the review of the medical records or data registration, although intra-observer variations cannot be excluded. The retrospective nature of the medical records bares the risk of overestimating disease counts by including outdated diagnoses. However, the chronicity of most of the conditions considered makes this a minor source of potential bias. On the other hand, there may be some diagnoses missing in the GPs’ documentation.

We found a high degree of multimorbidity among stroke survivors. There was no reason to believe that the patients in this study had particularly many co-morbidities compared with other stroke survivors. On the other hand, there were some reasons to assume the opposite. We excluded patients in nursing homes. It is a fair assumption that these were the patients with the greatest burden of disease.

The inclusion of patients started out wide. There was, however, a low degree of participation among invited patients. Possible explanations for this could be poor health and impaired physical and mental functioning among the patients. Impaired physical and mental functioning is associated with stroke as well as with multimorbidity [49–51]. It is therefore possible that the patients with the most complex multimorbidity were excluded in our study.

It may be regarded a weakness that the patient examples were hypothetical and not real patients. However, presenting real patient cases was deemed to risk the anonymity of the participants. Instead, a representative combination of conditions was strived for in the examples. The combinations of chronic conditions for the analysis were not influenced by the complexity of the relevant guidelines, i.e., there was no preference for conditions with comprehensive follow-up regimens. The criteria were that the condition was relatively frequent among the participants and that there should be specific guidelines for the condition. However, the combinations of conditions are to a large extent consistent with known patterns of co- and multimorbidity. Example 1 features the combination of stroke and COPD. The association between these conditions is previously described [52]. The association between stroke and RA in example 2 is also previously described [53, 54]. A disease cluster of cardiovascular diseases, metabolic diseases and mental health problems similar to that used in example 3, has previously been pointed out in a Norwegian population-based study on multimorbidity [18]. Associations between musculoskeletal problems and mental health problems and between musculoskeletal problems and cardiovascular problems was also identified in the same study [18].

Despite weaknesses, we claim the findings to be valid for the extent of multimorbidity among stroke survivors residing in the communities in this county.

## Conclusions

This study included stroke survivors residing in the communities. The GPs play a key role in the post-stroke follow-up of these patients. While guidelines for the follow-up exist, we have previously documented that adherence to these guidelines is weak [3]. In the present study, we have documented that all participants met the criteria for multimorbidity. Furthermore, we have demonstrated how adhering to the guidelines for each condition is a challenge, even for patients with few co-morbidities. For patients with more complex multimorbidity, adhering to the guidelines must be overwhelming and unmanageable for the GP. In this way, multimorbidity had a clear impact on the basis for the follow-up of patients with stroke in primary care. The findings provide new dimensions to the understanding of non-adherence to guidelines which should have implications for development of future guidelines.

## Supplementary information

**Additional file 1.** List of chronic conditions with operational definitions.

## Data Availability

Data could be available from the corresponding author on reasonable request.

## Abbreviations

BMI: Body mass index
BMRC: British Medical Research Council
CEA: Carcinoembryonic antigen
CEUS: Contrast-enhanced ultrasonography
COPD: Chronic obstructive pulmonary disease
DMARD: Disease modifying antirheumatic drug
GP: General practitioner
ICD-10: International Statistical Classification of Diseases and Related Health Problems, 10th revision
LDCT: Low-dose computed tomography
NR: No recommendations
RA: Rheumatoid arthritis
RGP: Regular general practitioner
SD: Standard deviation
