# Supplementary material for: Stroke follow-up in primary care: a Norwegian modelling study on the implications of multimorbidity for guideline adherence
Source: BMC Fam Pract. 2019 Oct 18;20:138. doi: 10.1186/s12875-019-1021-9 (PMC6798338; doi:10.1186/s12875-019-1021-9)
Supplement: Supplementary file 1 — Additional file 1. List of chronic conditions with operational definitions. [file 12875_2019_1021_MOESM1_ESM.docx]

**List of chronic conditions with operational definitions**

**Hypertension -** Read code ever recorded.

**Depression -** Read code recorded in last 12 months OR ≥4 anti-depressant prescriptions (excluding low dose tricyclics) in last 12 months.

**Painful condition -** ≥4 prescription only medicine analgesic prescriptions in last 12 months OR ≥4 specified anti-epileptics in the absence of an epilepsy Read code in last 12 months.

**Asthma (currently treated) -** Read code ever recorded AND any prescription in last 12 months.

**Coronary heart disease -** Read code ever recorded**.**

**Treated dyspepsia -** ≥4 prescriptions in last 12 months BNF 0103

**Diabetes -** Read code ever recorded.

**Thyroid disorders -** Read code ever recorded**.**

**Rheumatoid arthritis, other inflammatory polyarthropathies & systematic connective tissue disorders -** Read code ever recorded.

**Hearing loss -** Read code ever recorded**.**

**Chronic obstructive pulmonary disease -** Read code ever recorded**.**

**Anxiety & other neurotic, stress related & somatoform disorders -** Read code in last 12 months OR ≥4 anxiolytic/hypnotic prescriptions in last 12 months OR ≥4 10/25mg amitriptyline in last 12 months & do not meet the criteria for ‘Pain’.

**Irritable bowel syndrome -** Read code ever recorded OR ≥4 prescription only medicine antispasmodic prescription in last 12 months**.**

**New diagnosis of cancer in last five years -** Read code first recorded in last 5 years.

**Alcohol problems -** Read code ever recorded**.**

**Other psychoactive substance misuse -** Read code ever recorded.

**Treated constipation -** ≥4 laxative prescriptions in last year.

**Stroke & transient ischemic attack -** Read code ever recorded.

**Chronic kidney disease -** Read code ever recorded.

**Diverticular disease of intestine -** Read code ever recorded.

**Atrial fibrillation -** Read code ever recorded.

**Peripheral vascular disease** - Read code ever recorded.

**Heart failure** - Read code ever recorded.

**Prostate disorders** - Read code ever recorded.

**Glaucoma -** Read code ever recorded.

**Epilepsy** - Read code ever recorded AND antiepileptic prescription in last 12 months.

**Dementia** - Read code ever recorded.

**Schizophrenia (and related non-organic psychosis) or** **bipolar disorder** - Read code ever recorded/recorded in last 12 months (code dependent) OR Lithium prescribed in last 168 days.

**Psoriasis or eczema** - Read code ever recorded AND ≥4 related prescriptions in last 12 months (excluding simple emollients).

**Inflammatory bowel disease** - Read code ever recorded.

**Migraine -** ≥4 prescription only medicine anti-migraine prescriptions in last year.

**Blindness & low vision** - Read code ever recorded.

**Chronic sinusitis** - Read code ever recorded.

**Learning disability** - Read code ever recorded.

**Anorexia or bulimia** - Read code ever recorded.

**Bronchiectasis** - Read code ever recorded.

**Parkinson’s disease** - Read code ever recorded.

**Multiple sclerosis** - Read code ever recorded.

**Viral Hepatitis** - Read code ever recorded.

**Chronic liver disease** - Read code ever recorded.
